# Supplementary material for: Unveiling Neuroprotection and Regeneration Mechanisms in Optic Nerve Injury: Insight from Neural Progenitor Cell Therapy with Focus on Vps35 and Syntaxin12
Source: Cells. 2023 Oct 6;12(19):2412. doi: 10.3390/cells12192412 (PMC10572010; doi:10.3390/cells12192412)
Supplement: Supplementary file 1 [file cells-12-02412-s001.zip › supplementary figures.pptx]

## Slide 1
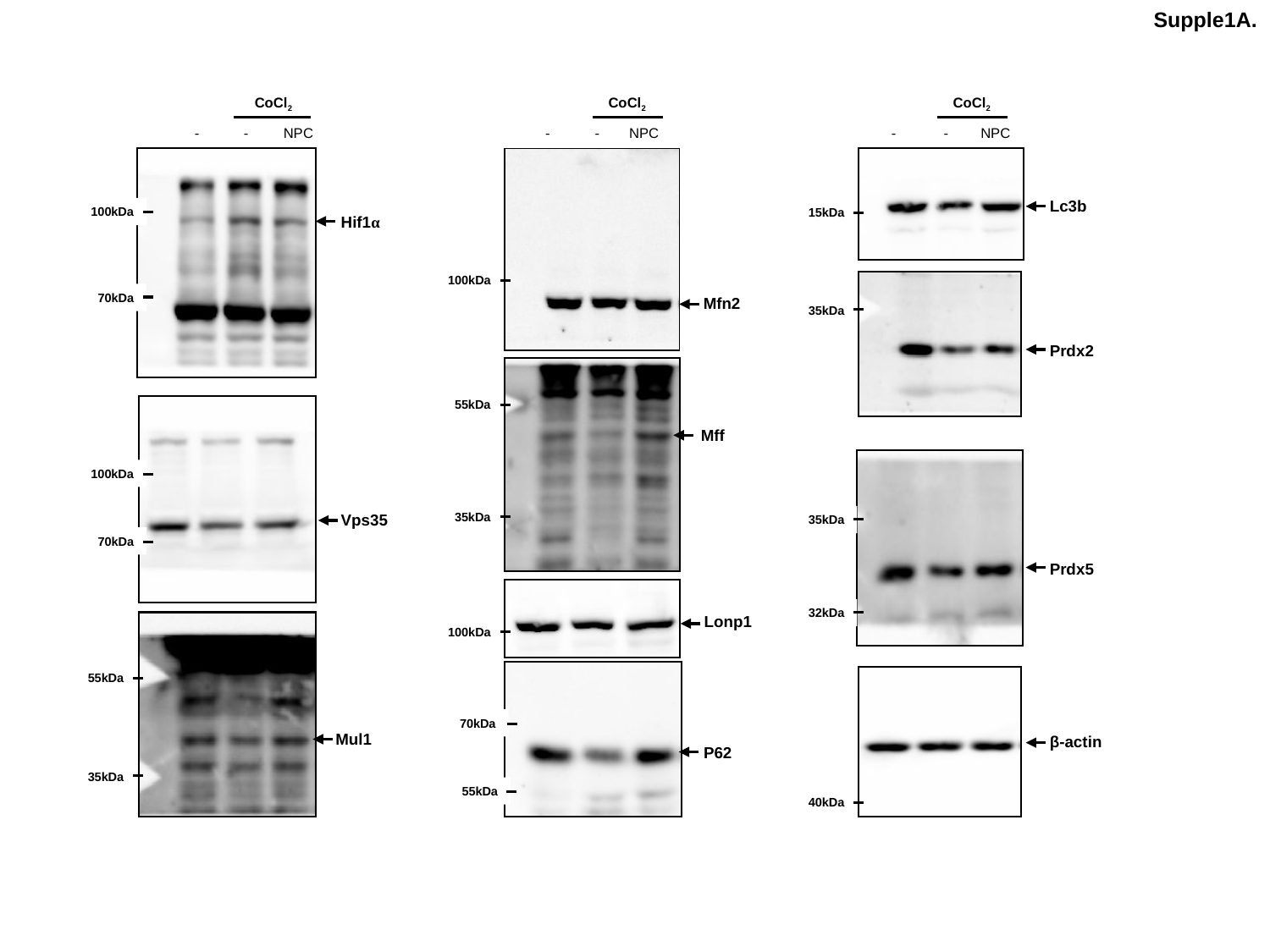

Supple1A.
CoCl2
CoCl2
CoCl2
| - | - | NPC |
| --- | --- | --- |
| - | - | NPC |
| --- | --- | --- |
| - | - | NPC |
| --- | --- | --- |
Lc3b
100kDa
15kDa
Hif1α
100kDa
70kDa
Mfn2
35kDa
Prdx2
55kDa
Mff
100kDa
35kDa
Vps35
35kDa
70kDa
Prdx5
32kDa
Lonp1
100kDa
55kDa
70kDa
Mul1
β-actin
P62
35kDa
55kDa
40kDa

## Slide 2
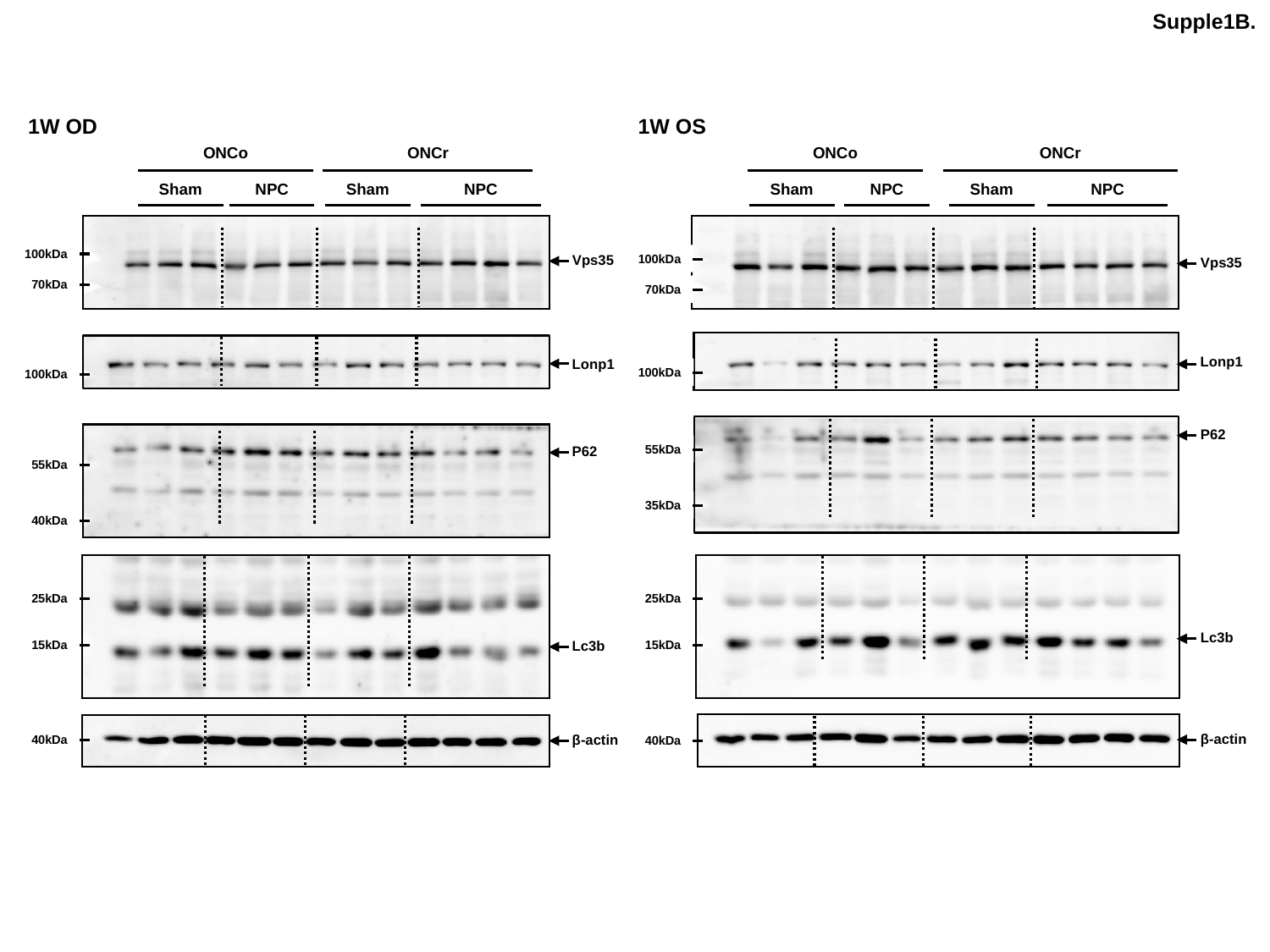

Supple1B.
1W OD
1W OS
ONCo
ONCr
ONCo
ONCr
Sham
NPC
Sham
NPC
Sham
NPC
Sham
NPC
100kDa
100kDa
Vps35
Vps35
70kDa
70kDa
Lonp1
Lonp1
100kDa
100kDa
P62
55kDa
P62
55kDa
35kDa
40kDa
25kDa
25kDa
Lc3b
15kDa
15kDa
Lc3b
β-actin
β-actin
40kDa
40kDa

## Slide 3
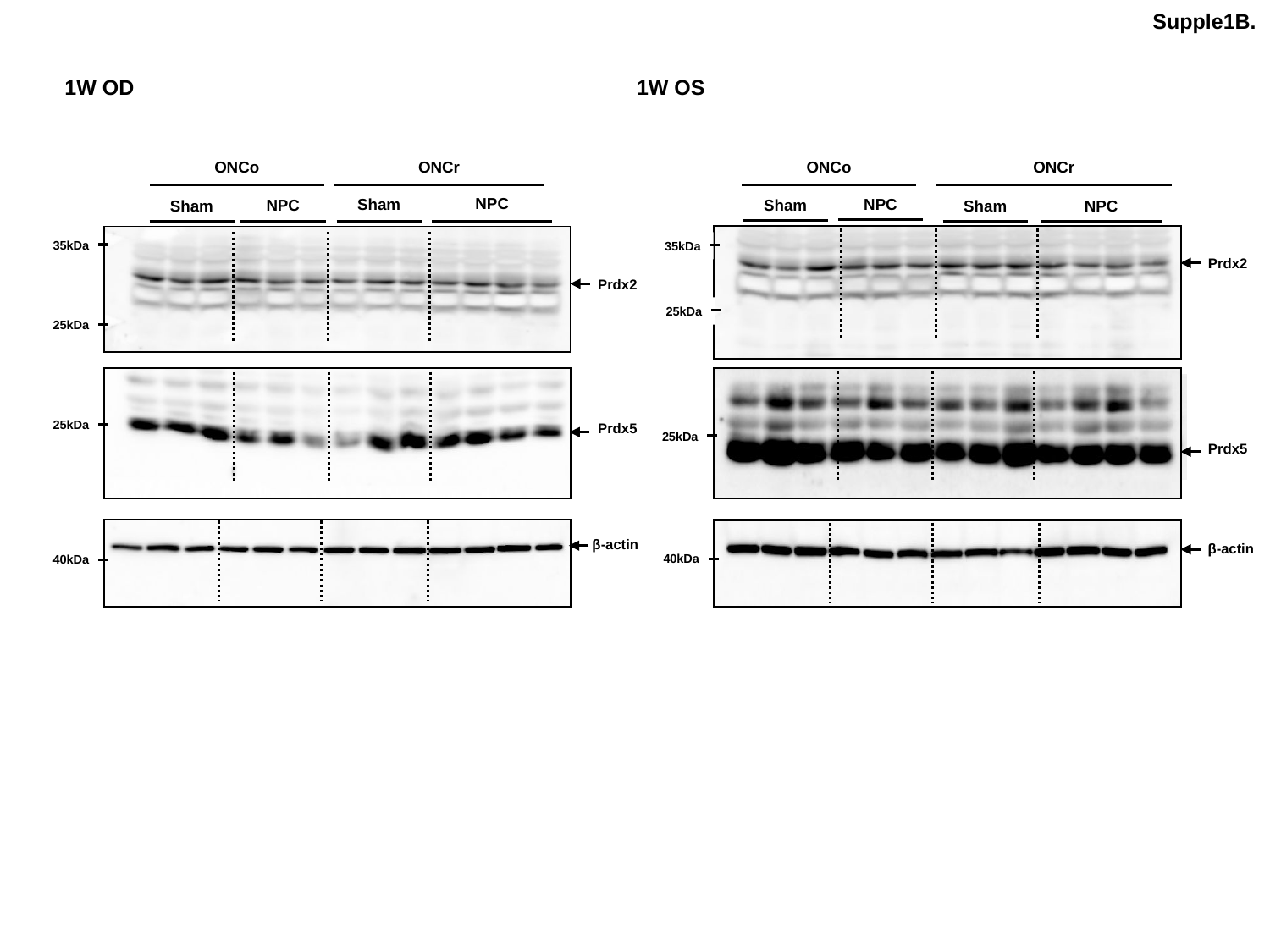

Supple1B.
1W OD
1W OS
ONCo
ONCr
ONCo
ONCr
NPC
Sham
NPC
NPC
Sham
Sham
Sham
NPC
35kDa
35kDa
Prdx2
Prdx2
25kDa
25kDa
25kDa
Prdx5
25kDa
Prdx5
β-actin
β-actin
40kDa
40kDa

## Slide 4
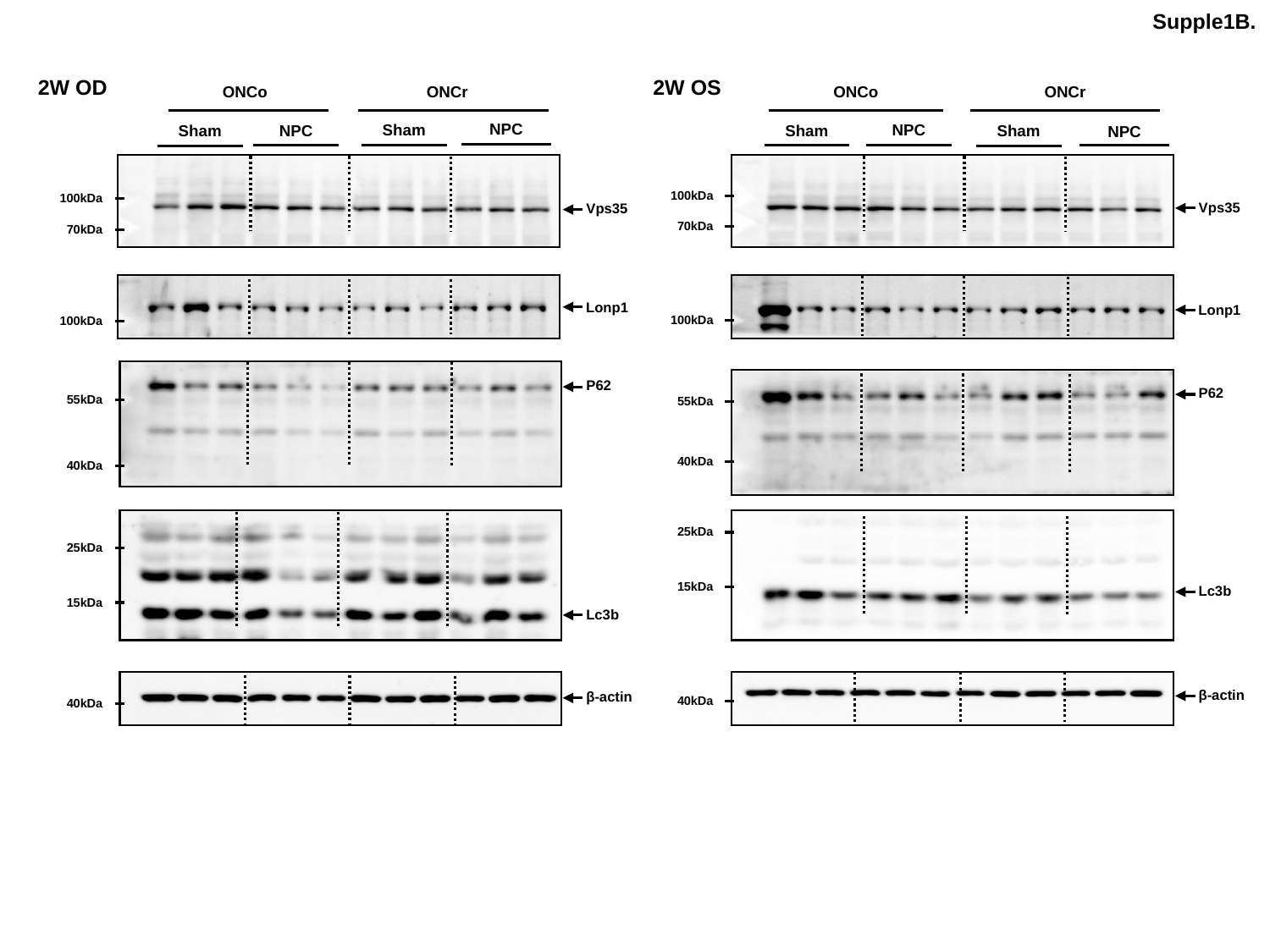

Supple1B.
2W OD
2W OS
ONCo
ONCr
ONCo
ONCr
NPC
Sham
NPC
NPC
Sham
Sham
Sham
NPC
100kDa
100kDa
Vps35
Vps35
70kDa
70kDa
Lonp1
Lonp1
100kDa
100kDa
P62
P62
55kDa
55kDa
40kDa
40kDa
25kDa
25kDa
15kDa
Lc3b
15kDa
Lc3b
β-actin
β-actin
40kDa
40kDa

## Slide 5
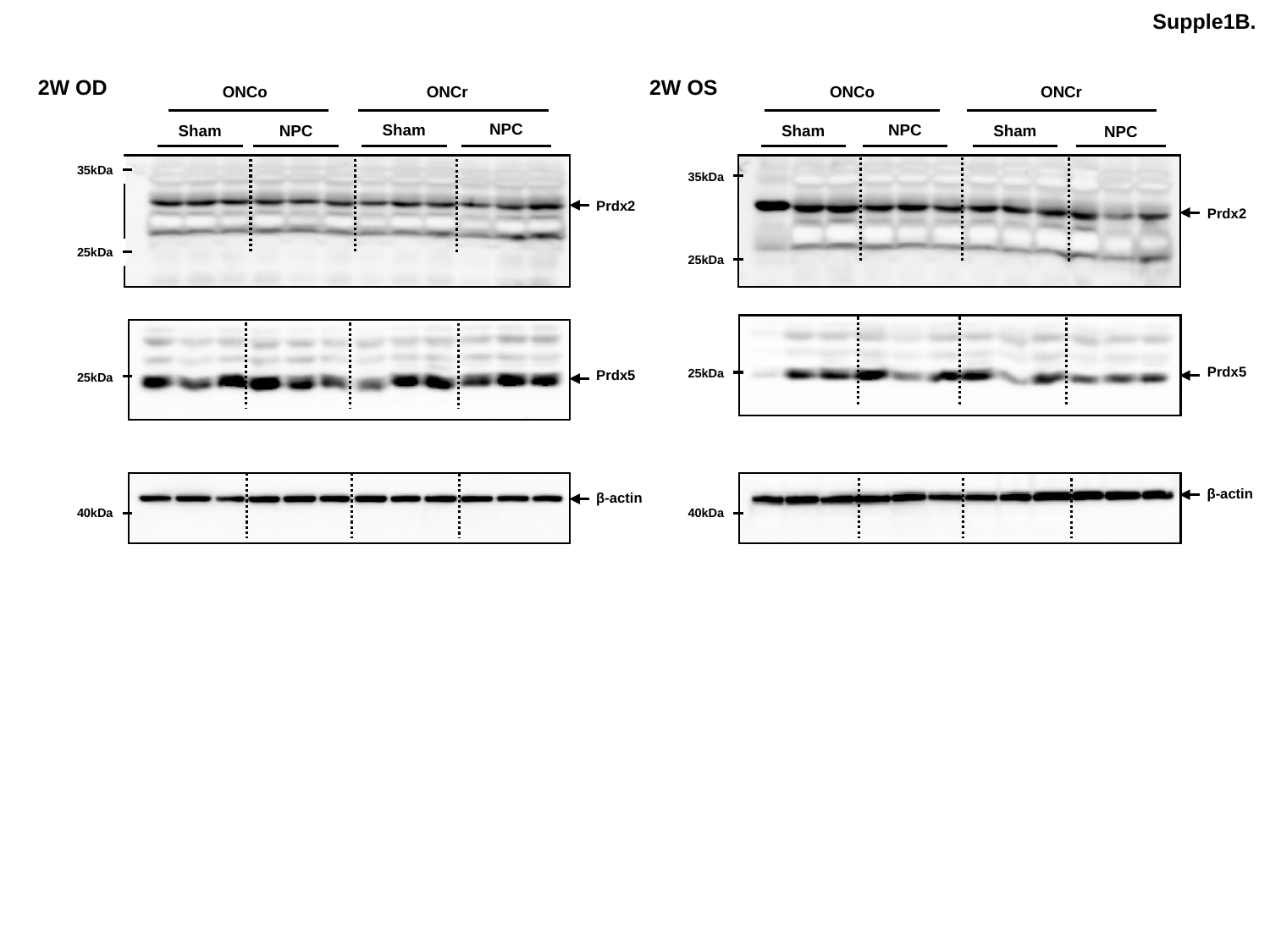

Supple1B.
2W OD
2W OS
ONCo
ONCr
ONCo
ONCr
NPC
Sham
NPC
NPC
Sham
Sham
Sham
NPC
35kDa
35kDa
Prdx2
Prdx2
25kDa
25kDa
Prdx5
25kDa
Prdx5
25kDa
β-actin
β-actin
40kDa
40kDa

## Slide 6
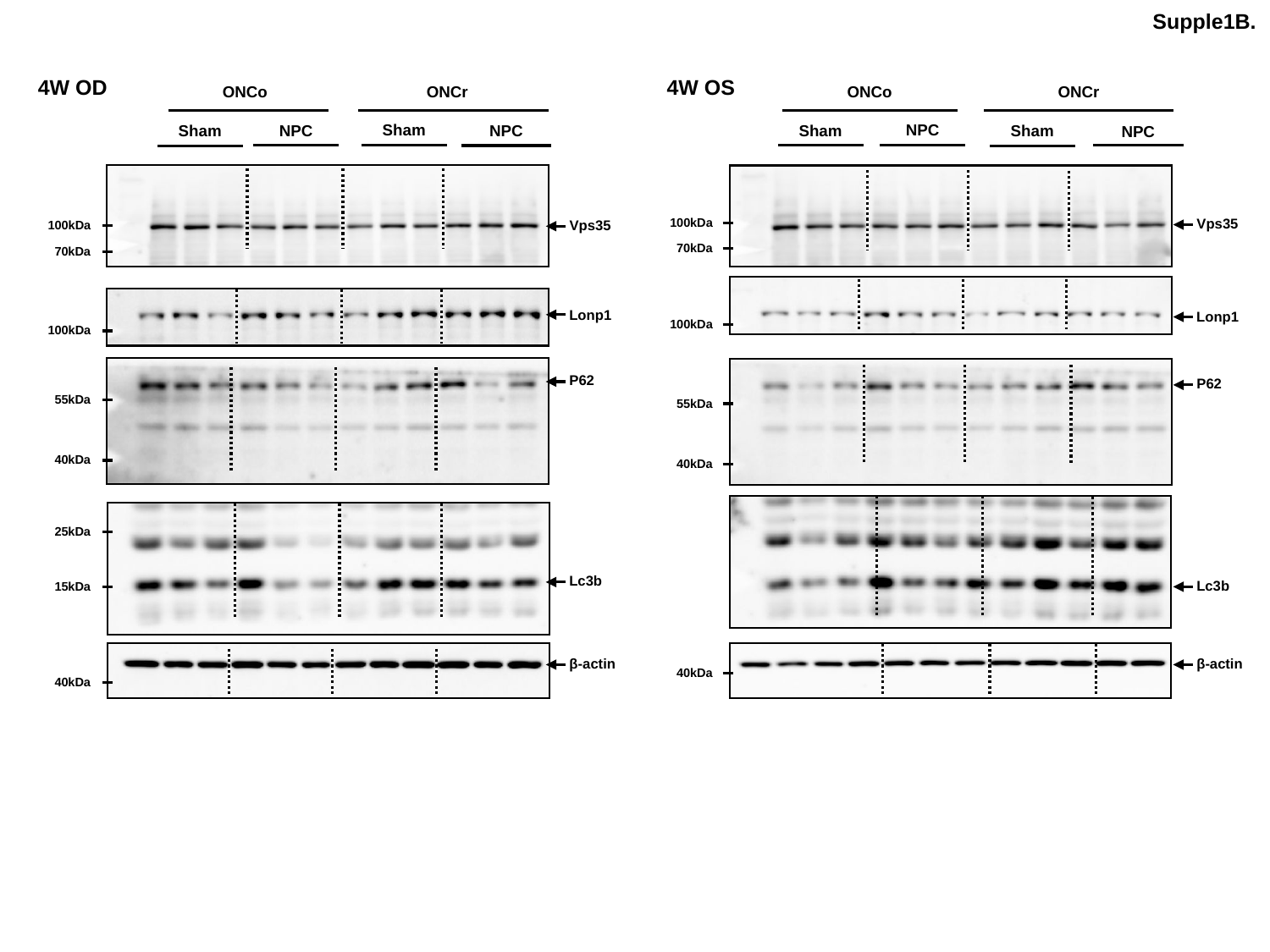

Supple1B.
4W OD
4W OS
ONCo
ONCr
ONCo
ONCr
Sham
NPC
NPC
Sham
NPC
Sham
Sham
NPC
Vps35
100kDa
Vps35
100kDa
70kDa
70kDa
Lonp1
Lonp1
100kDa
100kDa
P62
P62
55kDa
55kDa
40kDa
40kDa
25kDa
Lc3b
Lc3b
15kDa
β-actin
β-actin
40kDa
40kDa

## Slide 7
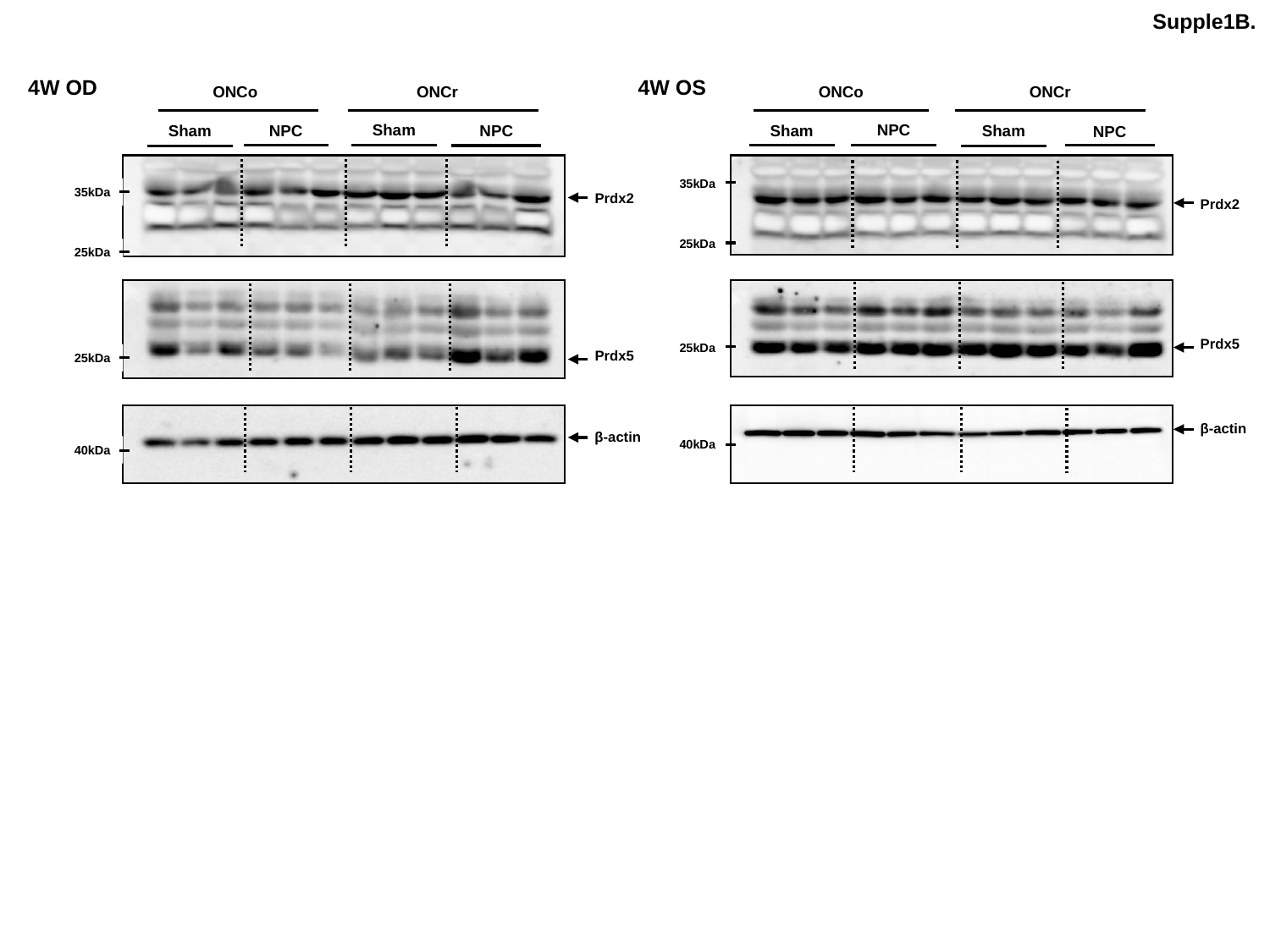

Supple1B.
4W OD
4W OS
ONCo
ONCr
ONCo
ONCr
Sham
NPC
NPC
Sham
NPC
Sham
Sham
NPC
35kDa
35kDa
Prdx2
Prdx2
25kDa
25kDa
Prdx5
25kDa
Prdx5
25kDa
β-actin
β-actin
40kDa
40kDa

## Slide 8
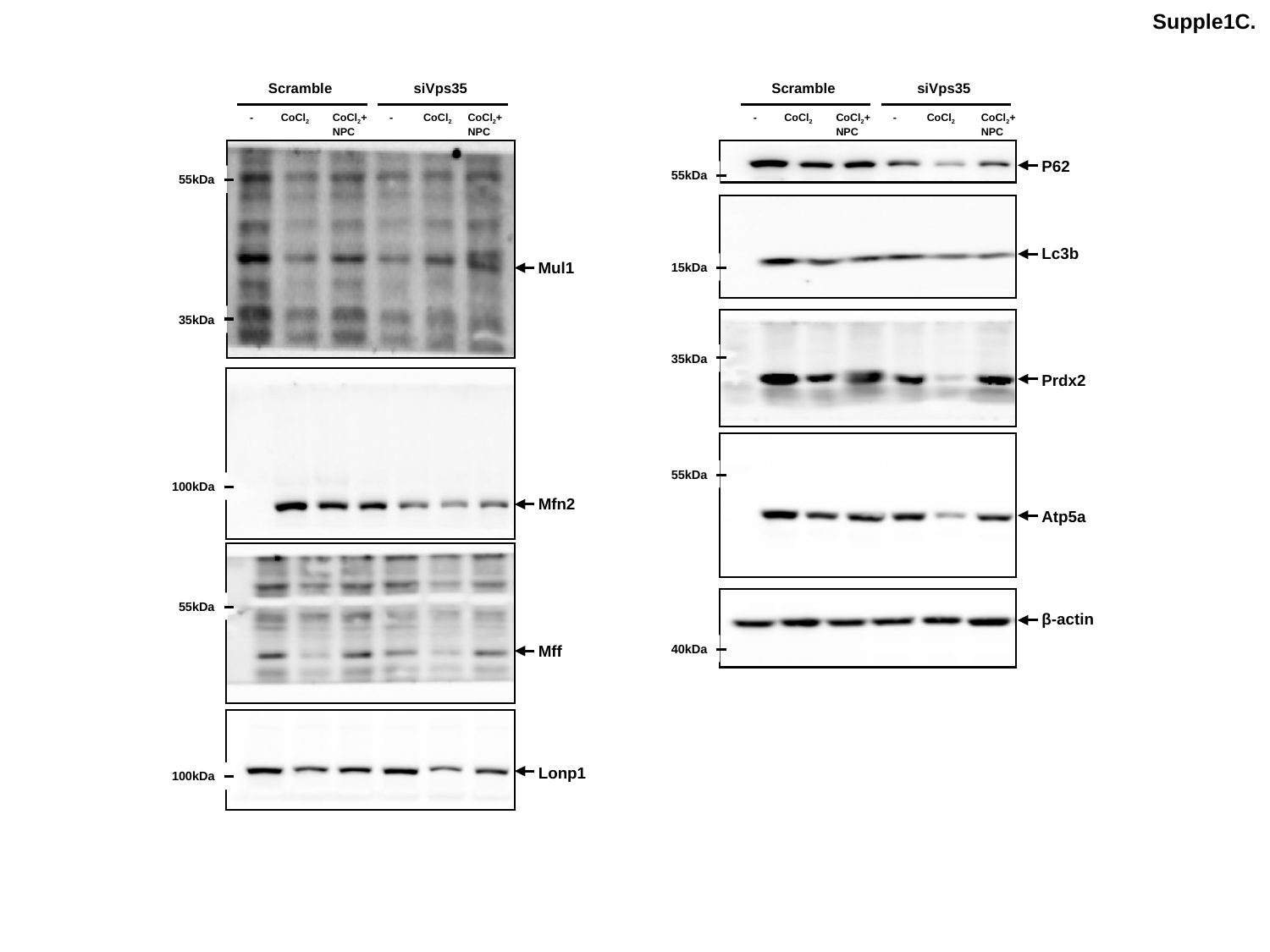

Supple1C.
Scramble
siVps35
Scramble
siVps35
-
CoCl2
CoCl2+
NPC
-
CoCl2
CoCl2+
NPC
-
CoCl2
CoCl2+
NPC
-
CoCl2
CoCl2+
NPC
P62
55kDa
55kDa
Lc3b
Mul1
15kDa
35kDa
35kDa
Prdx2
55kDa
100kDa
Mfn2
Atp5a
55kDa
β-actin
Mff
40kDa
Lonp1
100kDa

## Slide 9
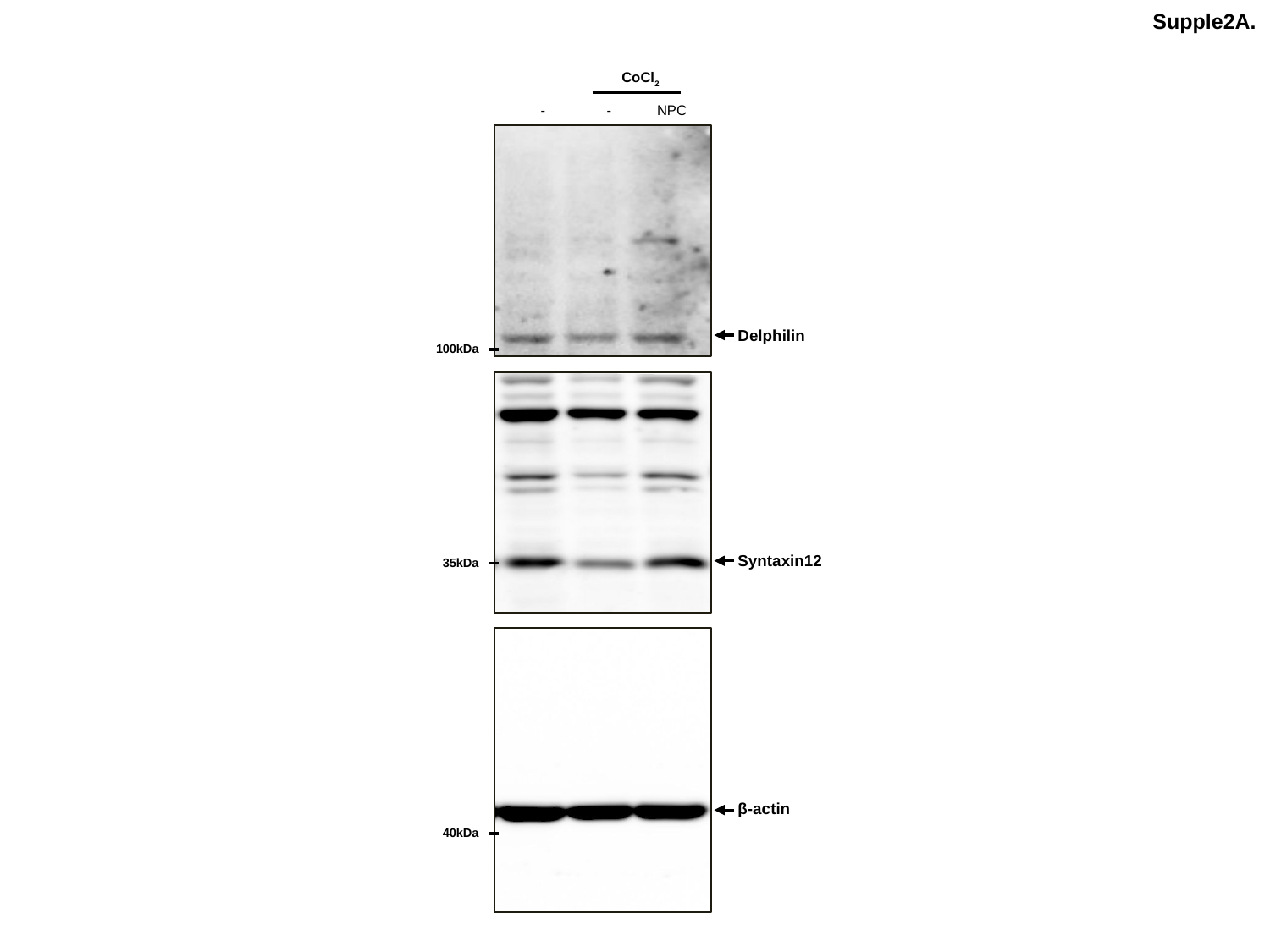

Supple2A.
CoCl2
| - | - | NPC |
| --- | --- | --- |
Delphilin
100kDa
Syntaxin12
35kDa
β-actin
40kDa

## Slide 10
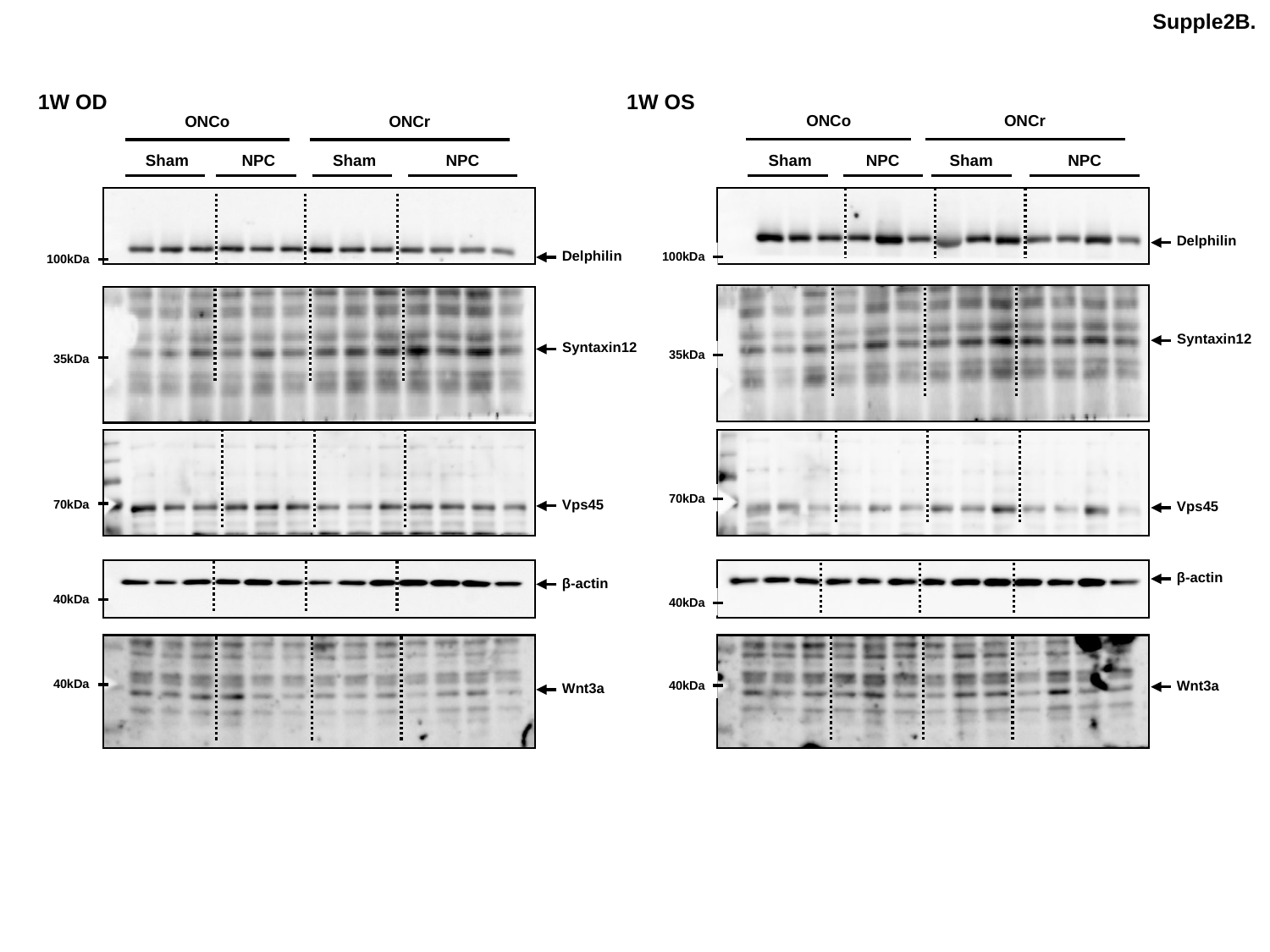

Supple2B.
1W OD
1W OS
ONCo
ONCr
ONCo
ONCr
Sham
NPC
Sham
NPC
Sham
NPC
Sham
NPC
Delphilin
Delphilin
100kDa
100kDa
Syntaxin12
Syntaxin12
35kDa
35kDa
70kDa
Vps45
70kDa
Vps45
β-actin
β-actin
40kDa
40kDa
40kDa
Wnt3a
40kDa
Wnt3a

## Slide 11
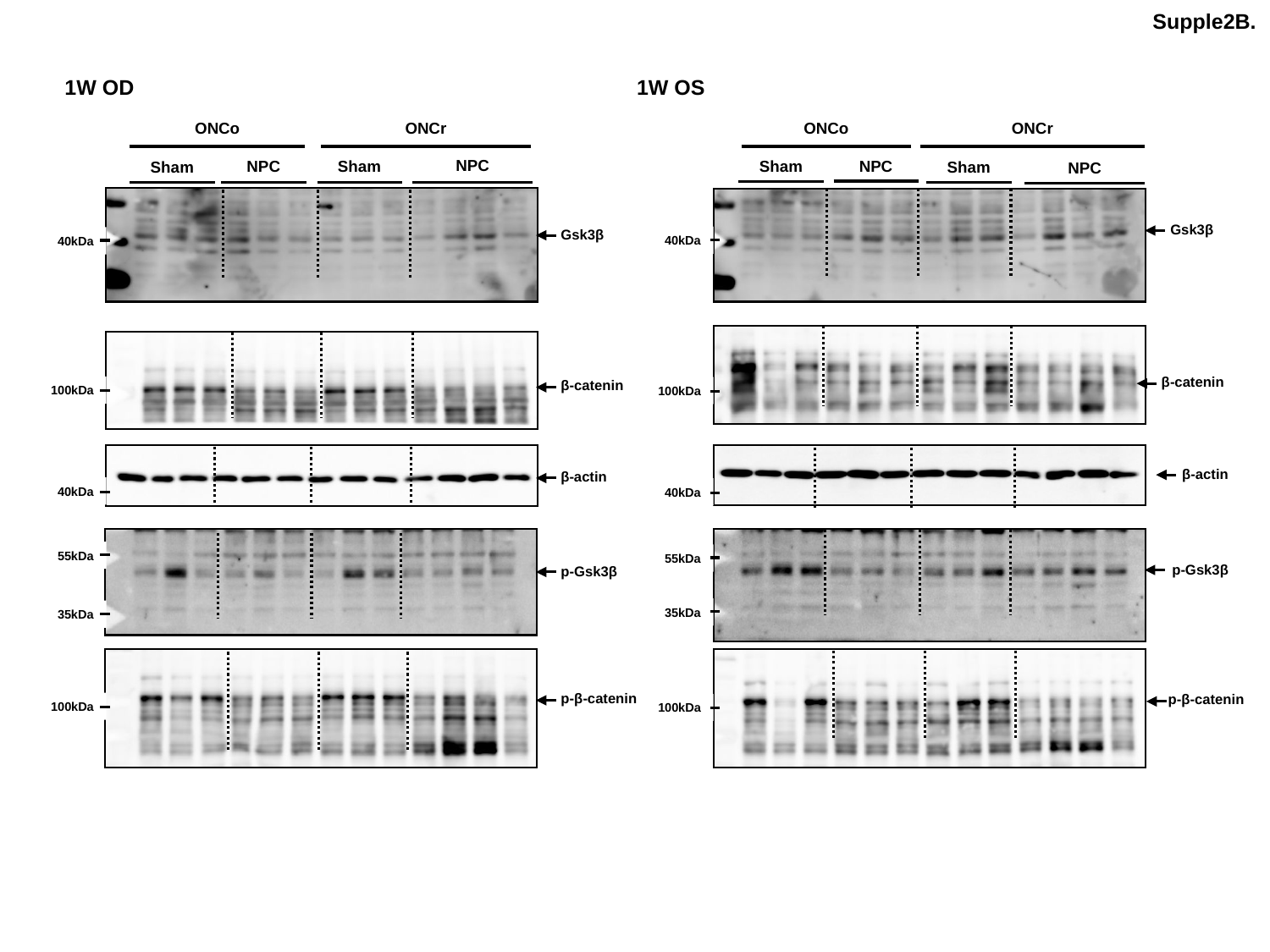

Supple2B.
1W OD
1W OS
ONCo
ONCr
ONCo
ONCr
NPC
Sham
NPC
NPC
Sham
Sham
Sham
NPC
Gsk3β
Gsk3β
40kDa
40kDa
β-catenin
β-catenin
100kDa
100kDa
β-actin
β-actin
40kDa
40kDa
55kDa
55kDa
p-Gsk3β
p-Gsk3β
35kDa
35kDa
p-β-catenin
p-β-catenin
100kDa
100kDa

## Slide 12
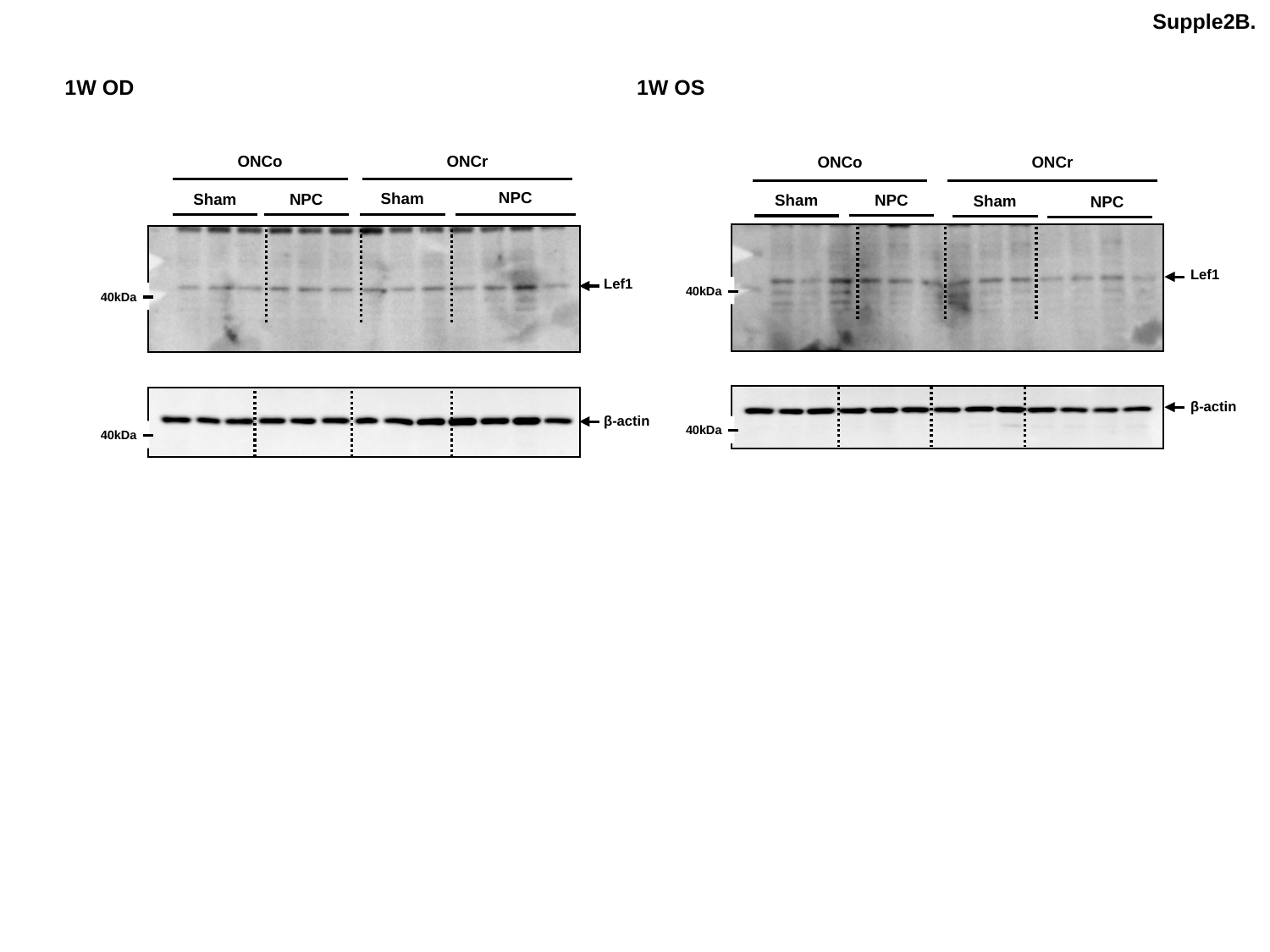

Supple2B.
1W OD
1W OS
ONCo
ONCr
ONCo
ONCr
NPC
Sham
NPC
Sham
NPC
Sham
Sham
NPC
Lef1
Lef1
40kDa
40kDa
β-actin
β-actin
40kDa
40kDa

## Slide 13
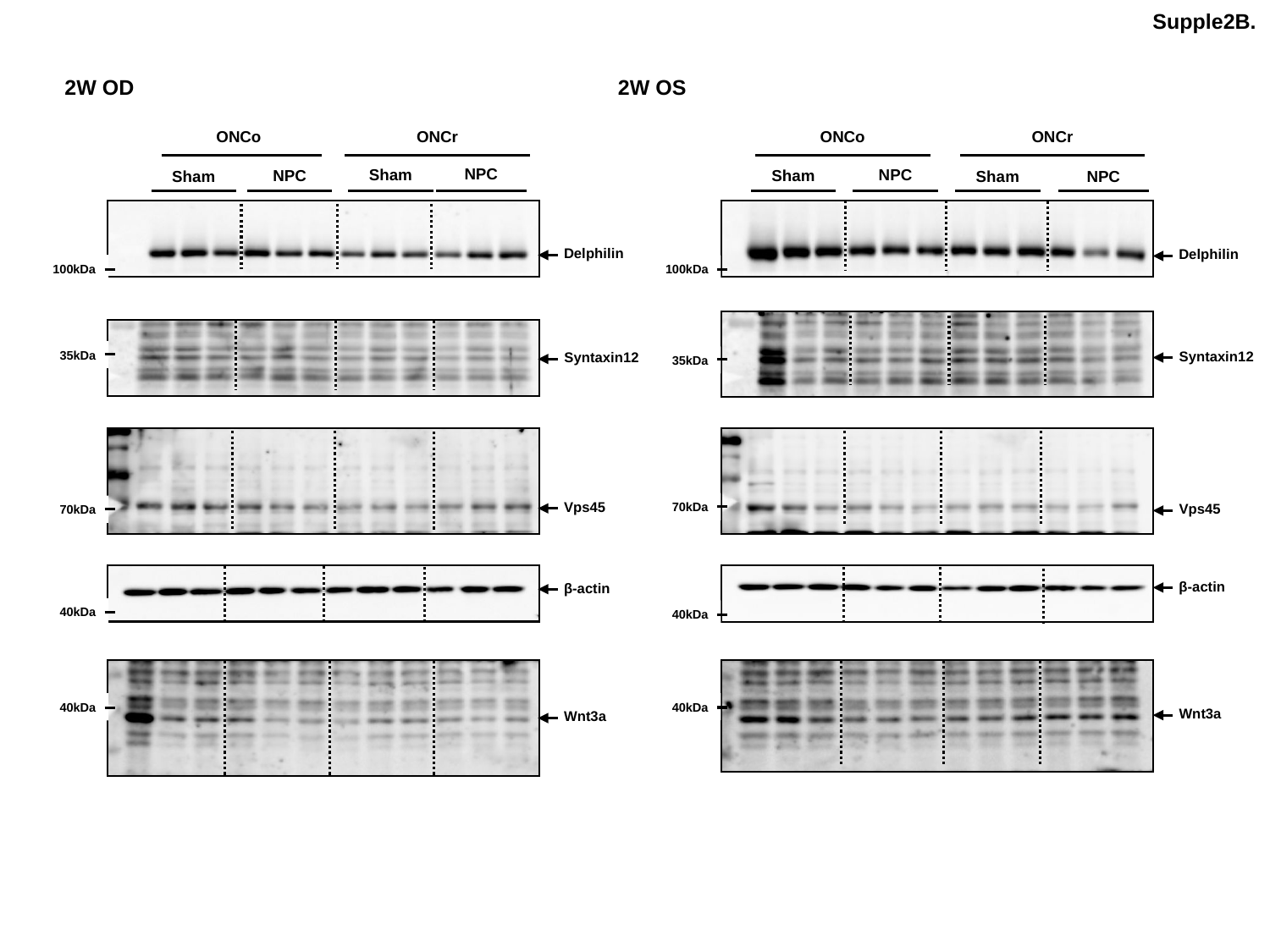

Supple2B.
2W OD
2W OS
ONCo
ONCr
ONCo
ONCr
NPC
Sham
NPC
NPC
Sham
Sham
Sham
NPC
Delphilin
Delphilin
100kDa
100kDa
35kDa
Syntaxin12
Syntaxin12
35kDa
Vps45
70kDa
Vps45
70kDa
β-actin
β-actin
40kDa
40kDa
40kDa
40kDa
Wnt3a
Wnt3a

## Slide 14
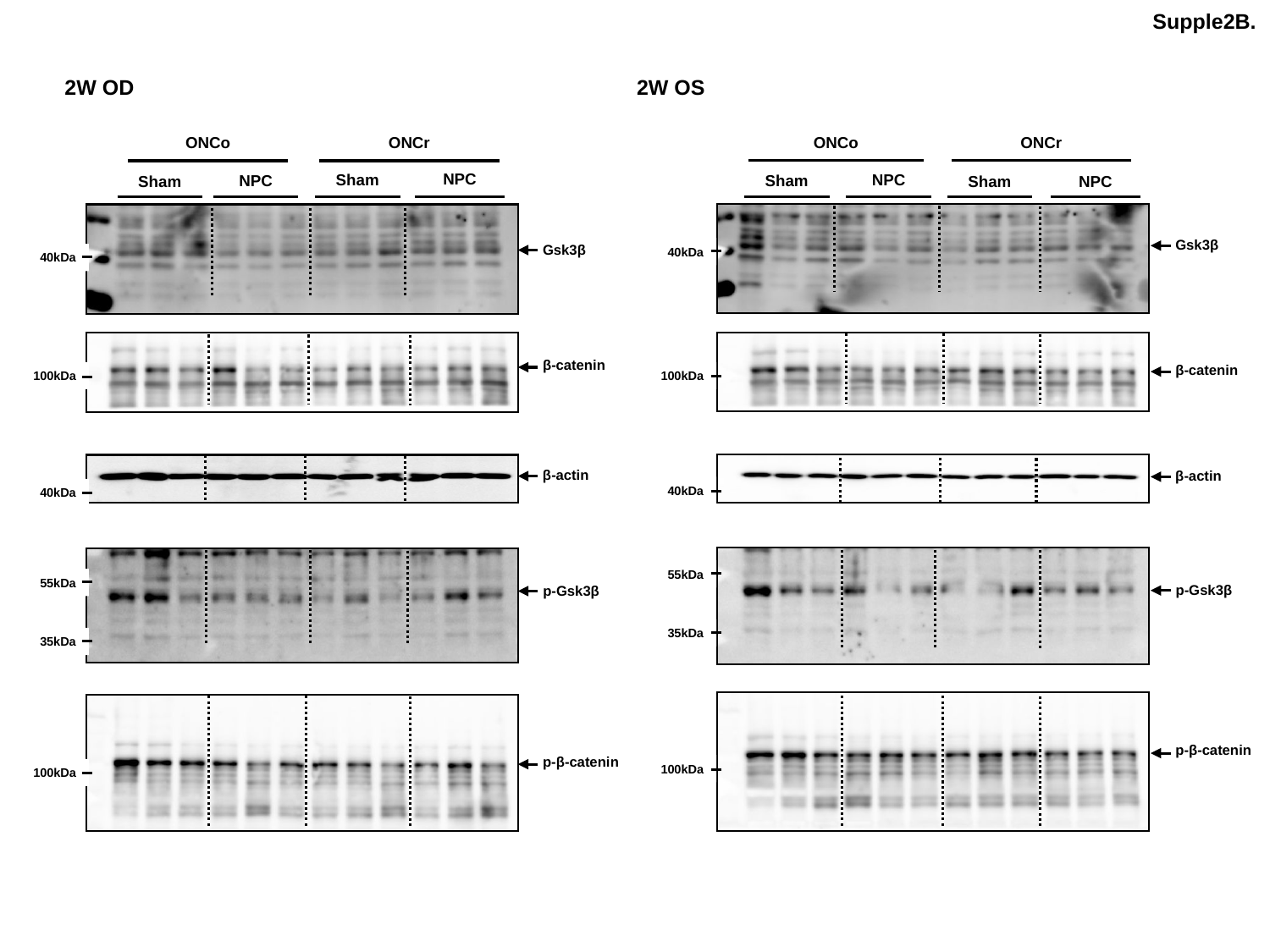

Supple2B.
2W OD
2W OS
ONCo
ONCr
ONCo
ONCr
NPC
NPC
Sham
Sham
NPC
Sham
Sham
NPC
Gsk3β
Gsk3β
40kDa
40kDa
β-catenin
β-catenin
100kDa
100kDa
β-actin
β-actin
40kDa
40kDa
55kDa
55kDa
p-Gsk3β
p-Gsk3β
35kDa
35kDa
p-β-catenin
p-β-catenin
100kDa
100kDa

## Slide 15
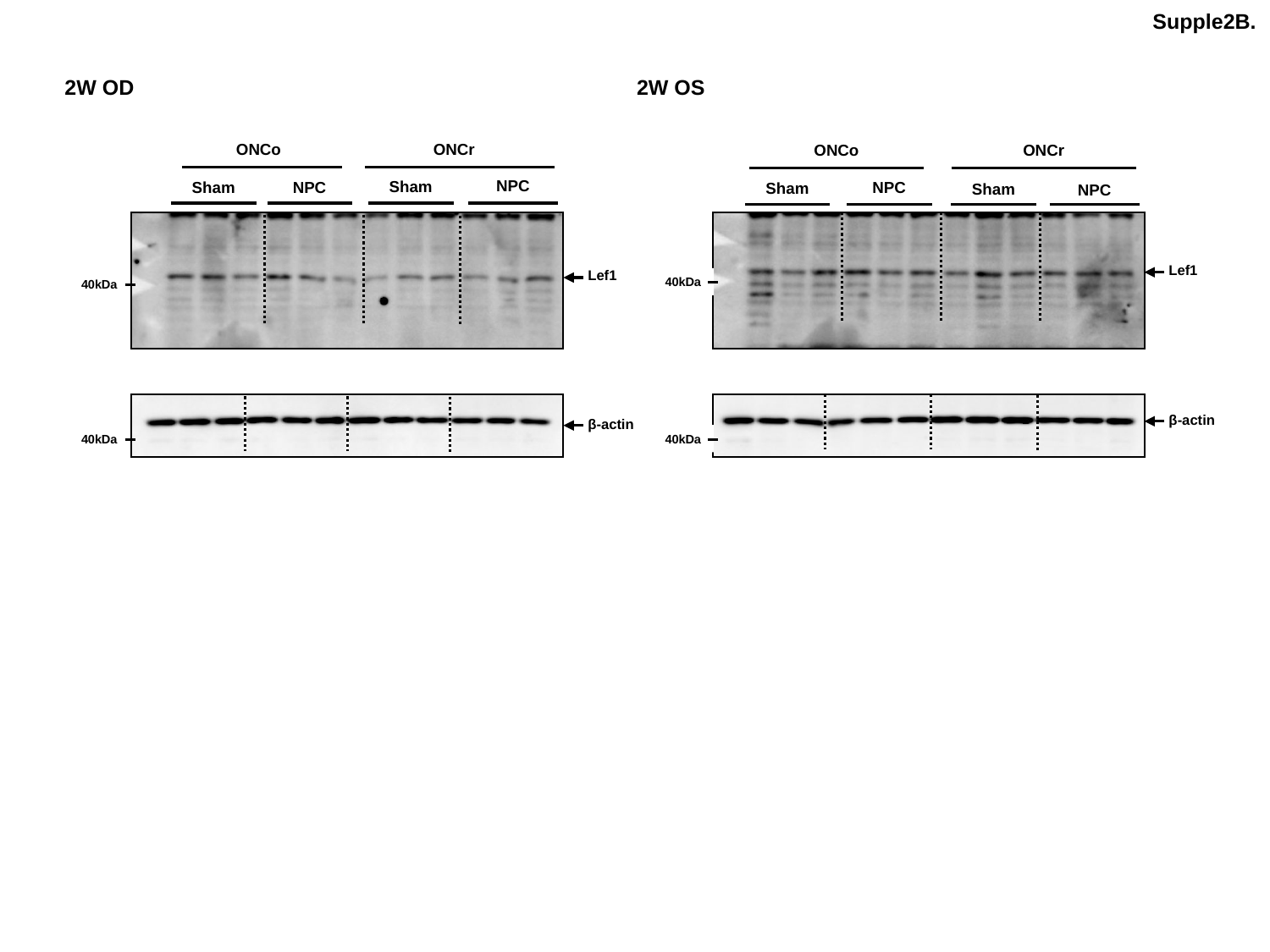

Supple2B.
2W OD
2W OS
ONCo
ONCr
ONCo
ONCr
NPC
Sham
NPC
NPC
Sham
Sham
Sham
NPC
Lef1
Lef1
40kDa
40kDa
β-actin
β-actin
40kDa
40kDa

## Slide 16
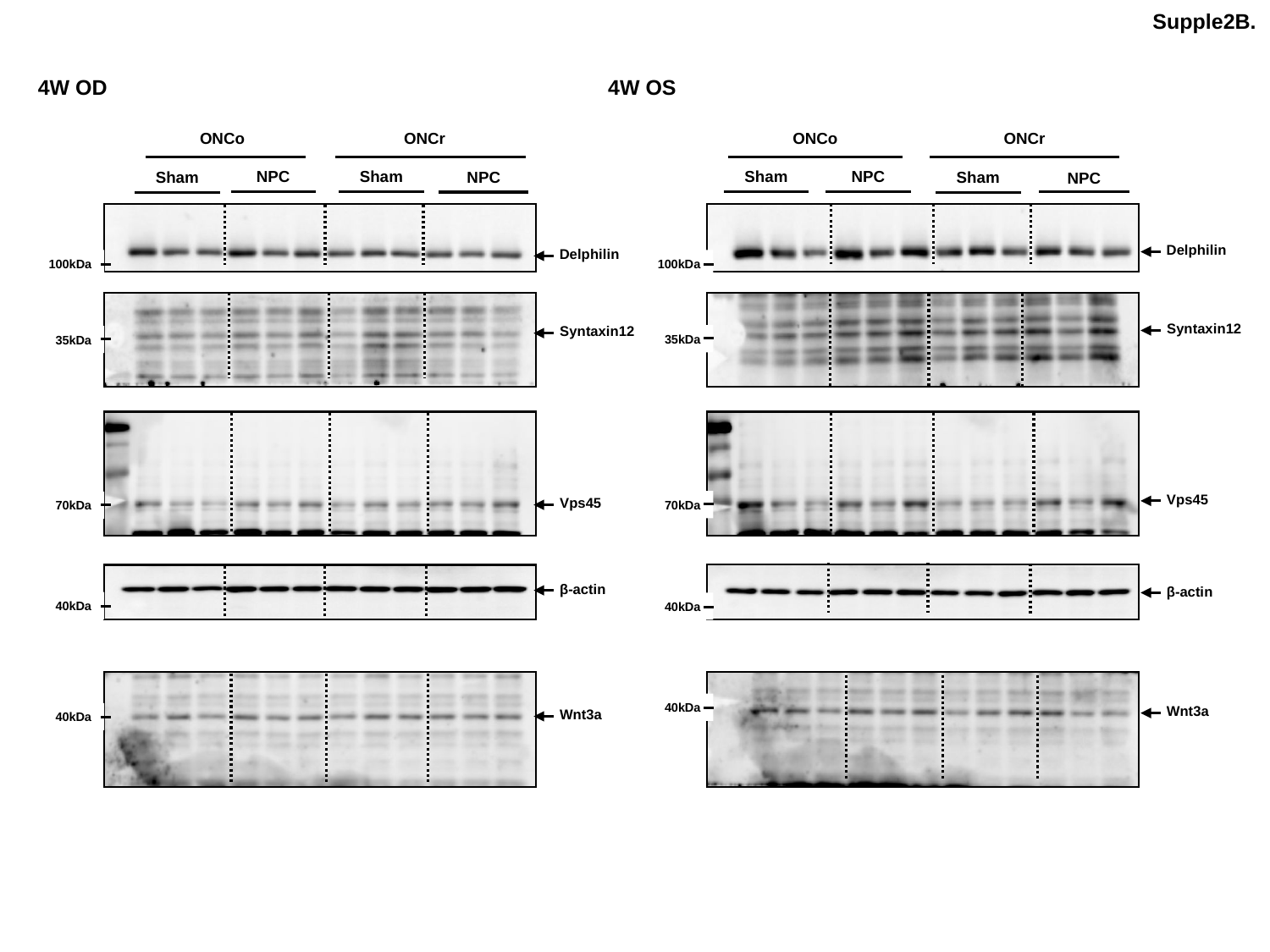

Supple2B.
4W OD
4W OS
ONCo
ONCr
ONCo
ONCr
Sham
NPC
NPC
Sham
NPC
Sham
Sham
NPC
Delphilin
Delphilin
100kDa
100kDa
Syntaxin12
Syntaxin12
35kDa
35kDa
Vps45
Vps45
70kDa
70kDa
β-actin
β-actin
40kDa
40kDa
40kDa
Wnt3a
Wnt3a
40kDa

## Slide 17
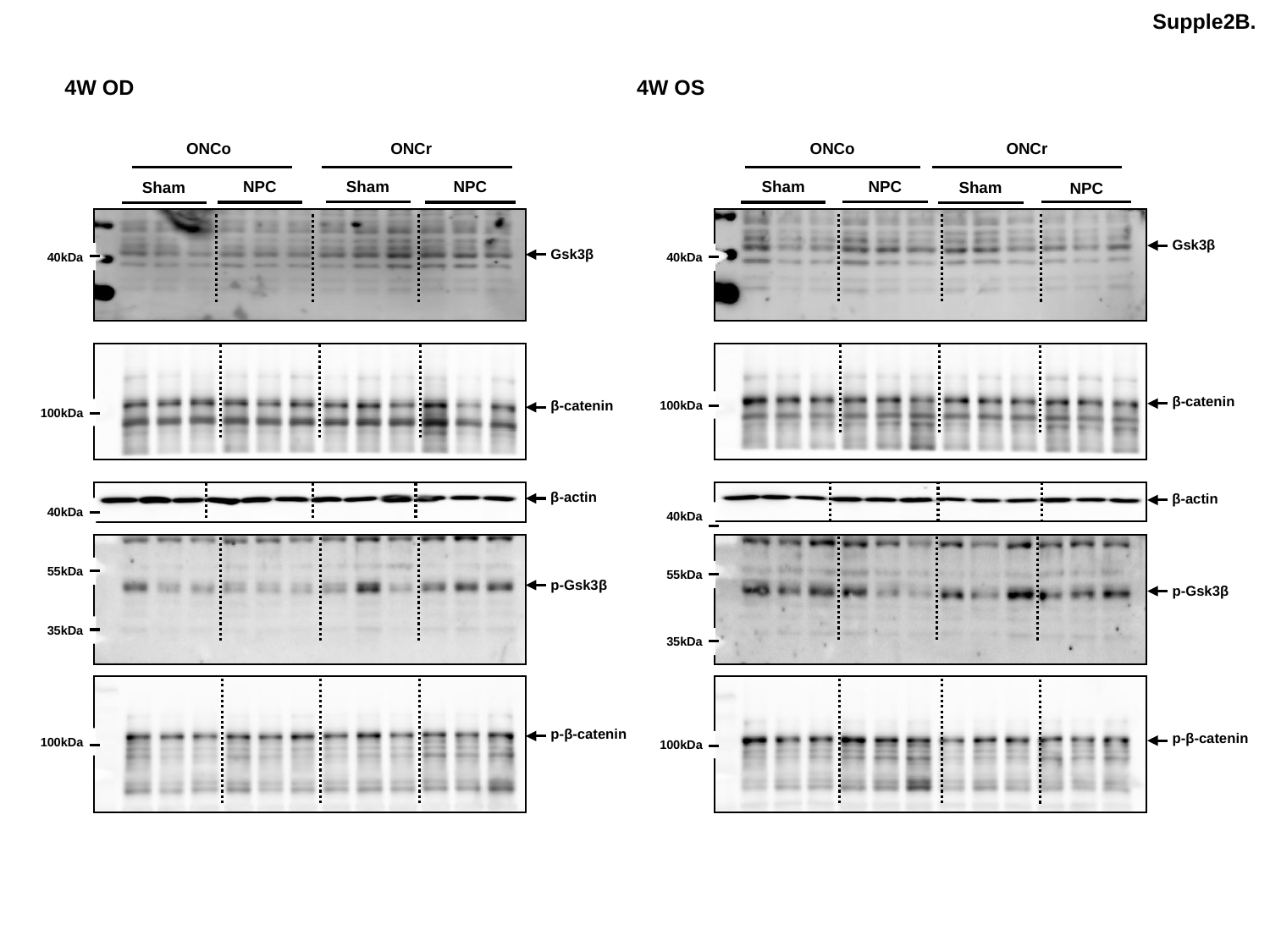

Supple2B.
4W OD
4W OS
ONCo
ONCr
ONCo
ONCr
Sham
NPC
NPC
Sham
NPC
Sham
Sham
NPC
Gsk3β
Gsk3β
40kDa
40kDa
β-catenin
β-catenin
100kDa
100kDa
β-actin
β-actin
40kDa
40kDa
55kDa
55kDa
p-Gsk3β
p-Gsk3β
35kDa
35kDa
p-β-catenin
p-β-catenin
100kDa
100kDa

## Slide 18
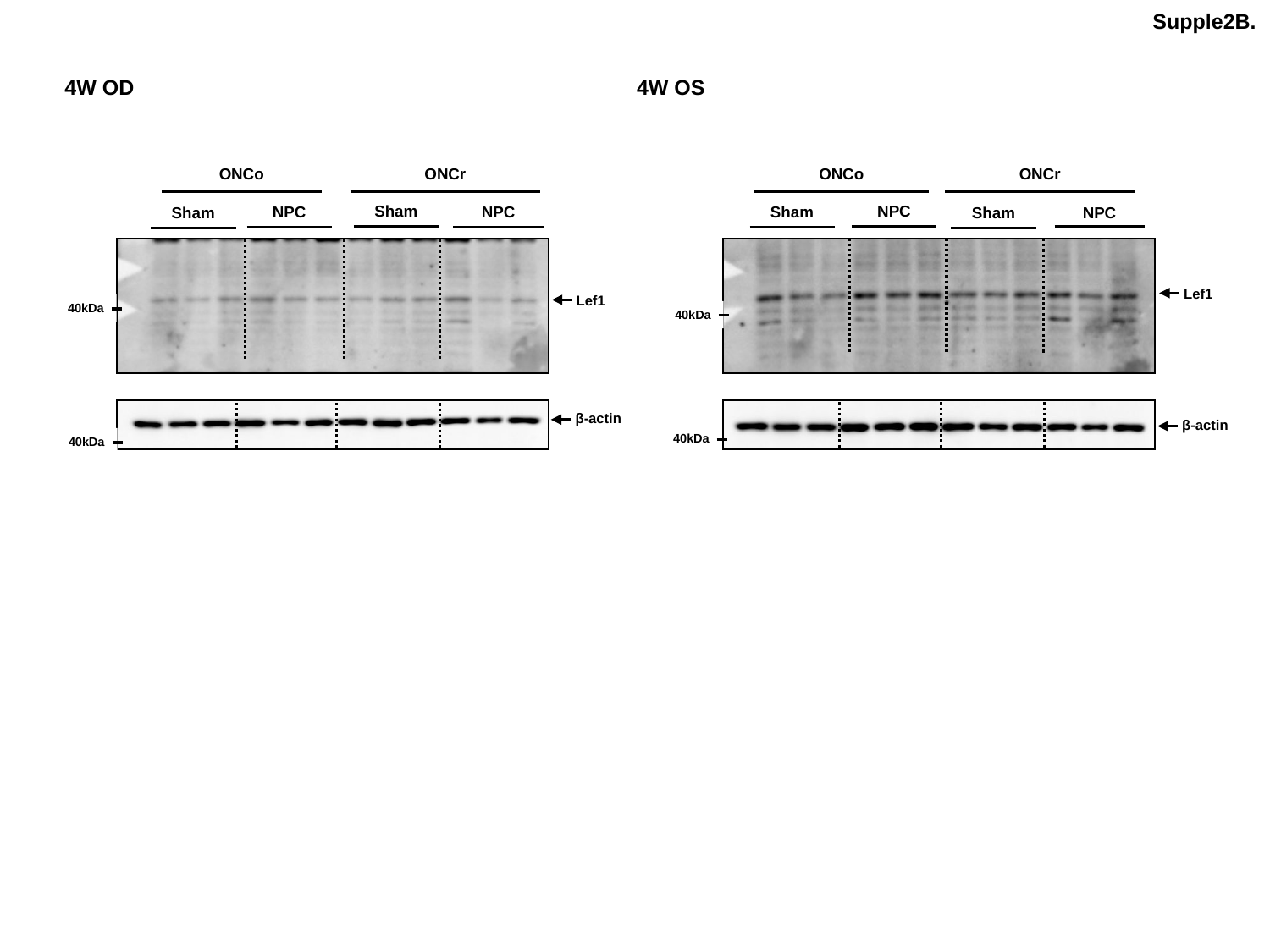

Supple2B.
4W OD
4W OS
ONCo
ONCr
ONCo
ONCr
Sham
NPC
NPC
Sham
NPC
Sham
Sham
NPC
Lef1
Lef1
40kDa
40kDa
β-actin
β-actin
40kDa
40kDa
