# Supplementary material for: Unveiling Neuroprotection and Regeneration Mechanisms in Optic Nerve Injury: Insight from Neural Progenitor Cell Therapy with Focus on Vps35 and Syntaxin12
Source: Cells. 2023 Oct 6;12(19):2412. doi: 10.3390/cells12192412 (PMC10572010; doi:10.3390/cells12192412)
Supplement: Supplementary file 1 [file cells-12-02412-s001.zip › Supplementary Table S1.pdf]

Table S1. The following tables include all the primary and secondary antibodies use in Immunoblot and Immunofluorescence (IFA) analysis.

Company	Primary antibody (Catalog Number)	Host	Application
Abcam, Cambridge, UK	Anti-Hifα (ab2185)	Rabbit	Immunoblot (1:1000)
	Anti-Gap43 (ab75810)	Rabbit	Immunoblot (1:1000)<br>IFA (1:200)
	Anti-Mul1 (ab209263)	Rabbit	Immunoblot (1:1000)
	Anti-p-β-catenin (ab11350)	Mouse	Immunoblot (1:1000)
	Anti-Lonp1 (ab224316)	Rabbit	Immunoblot (1:1000)
	Anti-Tuj1 (ab18207)	Rabbit	Immunoblot (1:1000)<br>IFA (1:200)
Genetex, CA, USA	Anti-Tnfα (GTX110520)	Rabbit	Immunoblot (1:1000)
	Anti-Vegf (GTX102643)	Rabbit	Immunoblot (1:1000)
	Anti-Wnt3a (GTX128101)	Rabbit	Immunoblot (1:1000)
	Anti-β-catenin (GTX101435)	Rabbit	Immunoblot (1:1000)
	Anti-Gsk3β (GTX111192)	Rabbit	Immunoblot (1:1000)
	Anti-Lef1 (GTX12034)	Mouse	Immunoblot (1:1000)
Santa Cruz, CA, USA	Anti-Thy1 (SC-53116)	Mouse	Immunoblot (1:200)
	Anti-Prdx5 (SC-133072)	Mouse	Immunoblot (1:1000)
	Anti-IL6 (SC-57315)	Mouse	Immunoblot (1:1000)
	Anti-β-actin (SC-47778)	Mouse	Immunoblot (1:1000)
	Anti-Delphilin (SC-390952)	Mouse	Immunoblot (1:1000)
	Anti-Atp5a (SC-136178)	Mouse	Immunoblot (1:1000)

Cell Signaling Technology, MA, USA	Anti-Nf (#2837)	Rabbit	Immunoblot (1:1000)
	Anti-Mfn2 (#9482)	Rabbit	Immunoblot (1:1000)
	Anti-Prdx2 (#46855)	Rabbit	Immunoblot (1:1000)
	Anti-p-Gsk3β (#9366)	Rabbit	Immunoblot (1:1000)
	Anti-Lc3b (#2775)	Rabbit	Immunoblot (1:1000)
	Anti-Gfap (#3670)	Mouse	Immunoblot (1:1000)<br>IFA (1:200)
Invitrogen, MA, USA	Anti-Iba1 (PA5-27436)	Rabbit	Immunoblot (1:1000)
	Anti-Vps35 (PA5-21898)	Rabbit	Immunoblot (1:1000)
	Anti-Bdnf (PA5-85730)	Rabbit	Immunoblot (1:1000)
Millipore, MA, USA	Anti-Brn3a (MAB1585)	Mouse	Immunoblot (1:1000)<br>IFA (1:25)
	Anti-NeuN (MAB377)	Mouse	Immunoblot (1:1000)
Proteintech, IL, USA	Anti-Syntaxin12 (14259-1-AP)	Rabbit	Immunoblot (1:1000)<br>IFA (1:50)
	Anti-Vps45 (12006-1-AP)	Rabbit	Immunoblot (1:1000)
Novus Biologicals, CO, USA	Anti-Nlrp3 (NBP2-12446)	Rabbit	Immunoblot (1:1000)
MyBioSource, CA, USA	Anti-Mff (MBS9126329)	Rabbit	Immunoblot (1:1000)
NSJ Bioreagents, CA, USA	Anti-P62 (R31058)	Rabbit	Immunoblot (1:1000)
